# IGFBP7 acts as a negative regulator of RANKL‐induced osteoclastogenesis and oestrogen deficiency‐induced bone loss

**DOI:** 10.1111/cpr.12752

**Published:** 2019-12-30

**Authors:** Chenyi Ye, Weiduo Hou, Mo Chen, Jinwei Lu, Erman Chen, Lan Tang, Kai Hang, Qianhai Ding, Yan Li, Wei Zhang, Rongxin He

**Affiliations:** ^1^ Department of Orthopedic Surgery the Second Affiliated Hospital School of Medicine Zhejiang University Hangzhou China; ^2^ Orthopedics Research Institute of Zhejiang University Hangzhou China; ^3^ Department of Rheumatology Second Affiliated Hospital School of Medicine Zhejiang University Hangzhou China

## Abstract

**Objectives:**

Insulin‐like growth factor‐binding protein 7 (IGFBP7) is a low‐affinity insulin growth factor (IGF) binder that may play an important role in bone metabolism. We previously reported that IGFBP7 enhanced osteogenic differentiation of bone marrow‐derived mesenchymal stem cells (BMSCs) via the Wnt/β‐catenin signalling pathway. In this study, we tried to reveal its function in osteoclast differentiation and osteoporosis.

**Methods:**

We used both in vitro and in vivo studies to investigate the effects of IGFBP7 on RANKL‐induced osteoclastogenesis and osteoporosis, together with the underlying molecular mechanisms of these processes.

**Results:**

We show that IGFBP7 inhibited receptor activation of nuclear factor‐κB (NF‐κB) ligand (RANKL)‐induced osteoclastogenesis, F‐actin ring formation and bone resorption, which was confirmed by using recombinant IGFBP7 protein, lentivirus and siRNA. The NF‐κB signalling pathway was inhibited during this process. Moreover, in a mouse ovariectomy‐induced osteoporosis model, IGFBP7 treatment attenuated osteoporotic bone loss by inhibiting osteoclast activity.

**Conclusions:**

Taken together, these findings show that IGFBP7 suppressed osteoclastogenesis in vitro and in vivo and suggest that IGFBP7 is a negative regulator of osteoclastogenesis and plays a protective role in osteoporosis. These novel insights into IGFBP7 may facilitate the development of potential treatment strategies for oestrogen deficiency‐induced osteoporosis and other osteoclast‐related disorders.

## 1 INTRODUCTION

Osteoporosis is one of the most common diseases in elderly individuals and is a devastating disease with significant mortality, morbidity and disability. Osteoporosis and osteopoenia affect approximately 72% of females and 62% of males over the age of 50 years.^1^, ^2^ Based on previous studies, there are more than 44 million Americans suffering from osteoporosis.^1^, ^3^ With the ageing population, there is an increasing prevalence of osteoporosis with subsequent fragility fractures, which continues to be a major challenge for orthopaedic surgeons and for society. Currently, the most common clinical treatment for osteoporosis is the use of medications such as bisphosphonates, calcitonin and oestrogen. However, due to widely reported serious side effects such as fever, hypocalcaemia, bone pain, hypercalcaemia, nausea, endometrial and breast cancer, and cardiovascular disease, these treatments are not optimal.^4^, ^5^ Identification of novel alternative treatment strategies with minimal side effects is therefore urgently needed.

Previous studies on postmenopausal osteoporosis showed that osteoporosis is mainly characterized by excessive osteoclastogenesis, abnormally increased osteoclastic bone resorption and deficient bone formation.^6^ Osteoclasts are usually differentiated from the monocyte/macrophage lineage upon stimulation with macrophage colony‐stimulating factor and receptor activator of nuclear factor‐κB ligand (RANKL). The interaction of RANKL with RANK results in the activation of intracellular downstream signalling pathways, including mitogen‐activated protein kinases (MAPKs), PI3K/AKT and NF‐κB. Following activation, transcription factors such as nuclear factor of activated T‐cell cytoplasmic 1 (NFATc1) and activator protein 1 are then activated, resulting in the induction of osteoclast markers including tartrate‐resistant acid phosphatase, matrix metalloproteinase 9 and cathepsin K, leading to the formation of mature multinucleated osteoclasts.^7^, ^8^ The mechanism of the disrupted balance of osteoblasts and osteoclasts is presently largely unknown, and there have been numerous efforts towards rebalancing this process. Because osteoclasts are the only cells capable of bone resorption, a better understanding of osteoclastogenesis, particularly the involvement of genetic factors, is necessary for the clinical management of osteoporosis.

Insulin‐like growth factor‐binding protein 7 (IGFBP7), also known as Mac25 or IGFBP‐related protein 1, is a secreted protein of the IGFBP superfamily, with binding affinity to insulin growth factor (IGF). Binding of IGFBP7 is much lower than that of conventional IGFBP1‐6. As a unique member of IGFBPs, studies have shown that IGFBP7 plays important roles in regulating cell proliferation, adhesion and differentiation in many cell lines.^9^, ^10^ IGFBP7 has also been implicated as a tumour suppressor in a variety of human malignancies including thyroid carcinoma,^11^ cholangiocarcinoma,^12^ gastric cancer,^13^ hepatocellular carcinoma^14^ and breast cancer.^15^ Notably, there is increasing evidence, suggesting that IGFBP7 is involved in bone metabolism. Pereira et al found that the expression of IGFBP7 and parathyroid hormone (PTH) was increased in osteoblasts.^16^ Arnold et al reported that IGFBP7 promoted osteogenesis of stem cells and may protect them from bone disease in multiple myeloma.^17^ Using protein interaction and microRNA network analyses, Wang et al reported that IGFBP7 expression was associated with the development of osteoarthritis (OA),^18^ which was confirmed by two other studies.^19^, ^20^ In our previous study, we reported that IGFBP7 enhanced osteogenic differentiation of BMSCs via the Wnt/β‐catenin signalling pathway in vitro and promoted new bone formation in vivo.^21^ However, the function of IGFBP7 in osteoclast differentiation and osteoporosis still remains unclear.

For a better understanding of the role of IGFBP7 in bone metabolism, we used both in vitro and in vivo studies to investigate the effects of IGFBP7 on RANKL‐induced osteoclastogenesis and osteoporosis and characterized the underlying molecular mechanisms of these processes.

## 2 MATERIALS AND METHODS

### 2.1 Reagents

Alpha modification of Eagle's medium (α‐MEM), PBS and 1% penicillin/streptomycin (P/S) were purchased from GENOM. Foetal bovine serum (FBS) was purchased from Gemini. Recombinant M‐CSF and RANKL were purchased from Novoprotein. Cell Counting Kit‐8 (CCK‐8) was purchased from Dojindo (Kumamoto, Japan). Antibodies against p38, phospho‐p38 (p‐p38), c‐Jun N‐terminal kinase (JNK), p‐JNK, p65, p‐p65, extracellular signal‐regulated kinase (ERK), p‐ERK, nuclear factor of kappa light polypeptide gene enhancer in B‐cell inhibitor alpha (IκBα), p‐IκBα, AKT, p‐AKT, osteocalcin (OCN), transforming growth factor β‐activated kinase 1 (TAK1), p‐TAK1 and glyceraldehyde‐3‐phosphate dehydrogenase (GAPDH) were purchased from Cell Signaling Technology (CST). Antibodies against NFATc1/NFAT2 and c‐Fos were purchased from Abcam. Antibodies against OPG and RANKL were purchased from Affinity Biosciences (Changzhou, China). HRP‐linked secondary antibodies against IgG were purchased from BOSTER Biological Technology. About 4% paraformaldehyde (PFA) and the TRAP staining kit were purchased from Sigma‐Aldrich. Masson's trichrome staining kit was obtained from Nanjing Jiancheng Bioengineering Institute. Alkaline phosphatase (ALP) activity kit was supplied by Beyotime. RNAiso reagent, complementary DNA (cDNA) synthesis kit and SYBR Premix Ex Taq™ II kit were purchased from TaKaRa. Recombinant IGFBP7 protein was purchased from R&D Systems.

### 2.2 Bone marrow‐derived macrophage (BMM) preparation and osteoclast differentiation

Bone marrow‐derived macrophages were prepared from long bones of 6‐ to 8‐week‐old male C57BL/6 mice as previously described,^7^ which were then cultured in complete α‐MEM supplemented with 10% FBS, 1% P/S and 30 ng/mL M‐CSF. After that, BMMs (8 × 10^3 ^cells/well) were seeded into 96‐well plates and cultured with M‐CSF (30 ng/mL), RANKL (100 ng/mL) and different concentrations of recombinant IGFBP7 protein (0‐1000 ng/mL) for 5 days until the formation of mature osteoclasts. After that, cells were fixed with 4% PFA and stained with a TRAP staining kit, following the standard procedures. TRAP‐positive cells with five or more nuclei were considered as mature osteoclasts, which were then counted and quantified using ImageJ software (NIH).

### 2.3 Cell viability and cytotoxicity assay

To assess the effect of IGFBP7 on BMM viability, BMMs (8 × 10^3 ^cells/well) were seeded into a 96‐well plate and cultured in α‐MEM containing 30 ng/mL M‐CSF and serial concentration of recombinant IGFBP7 protein (0‐1000 ng/mL) for 48 or 96 hours. Then, the medium was removed and BMMs were treated with 100 μL α‐MEM containing M‐CSF (30 ng/mL) and 10% CCK‐8 for another 4 hours at 37°C. The optical density (OD) value at 450 nm was measured by a microplate reader (ELX808; BioTek).

### 2.4 RNA extraction, reverse transcription and real‐time PCR

Total RNA from BMMs or RAW264.7 cells was extracted using RNAiso reagent (TaKaRa) and quantified by reading the absorbance at 260 nm (NanoDrop 2000; Thermo Fisher). According to the manufacturer's instructions (Takara), cDNA was synthesized using total RNA (≤1000 ng) in a reaction volume of 20 μL. After that, RT‐PCR was then performed on the ABI StepOnePlus System (Applied Biosystems) using SYBR Premix Ex Taq^™^ II kit (Takara), following cycling conditions as: 5°C for 30 seconds and 45 cycles of denaturation at 95°C for 5 seconds and amplification at 60°C for 30 seconds. The 2^−△△Ct^ method was used to calculate the relative expression levels of target genes. The detailed murine primer sequences of all genes are shown in Table 1. 18S was amplified as a housekeeping gene.

**Table 1:** Sequences of primers for quantitative real‐time PCR

Gene	Reverse (5′‐3′)	Reverse (3′‐5′)
IGFBP7	TCAGCGGACAGAACTCTTGC	CCAGCCCGTTACTTCATGCT
NFATc1	GGAGAGTCCGAGAATCGAGAT	TTGCAGCTAGGAAGTACGTCT
TRAP	CACTCCCACCCTGAGATTTGT	CCCCAGAGACATGATGAAGTCA
V‐ATPase‐d2	CAGAGCTGTACTTCAATGTGGAC	AGGTCTCACACTGCACTAGGT
CTSK	CTCGGCGTTTAATTTGGGAGA	TCGAGAGGGAGGTATTCTGAGT
DC‐STAMP	GGGGACTTATGTGTTTCCACG	ACAAAGCAACAGACTCCCAAAT
MMP‐9	CTGGACAGCCAGACACTAAAG	CTCGCGGCAAGTCTTCAGAG
IGF1R	TGACATCCGCAACGACTATCA	CCAGTGCGTAGTTGTAGAAGAGT
18S	CGGACACGGACAGGATTGACA	CCAGACAAATCGCTCCACCAACTA

### 2.5 Bone resorption pit assay

To explore the effects of IGFBP7 on osteoclast function, bone resorption pit assay was performed using the standard procedure. In brief, BMMs (3.2 × 10^4 ^cells/well) were seeded onto bovine bone slides in 24‐well plates. After 24 hours, cells were treated with RANKL (100 ng/mL) and M‐CSF (30 ng/mL) and different concentrations of recombinant IGFBP7 (0, 250, 500 and 1000 ng/mL) for 5 days. Using the same method, RAW264.7 cells (6 × 10^3 ^cells/well) were also seeded onto bovine bone slides in 24‐well plates and treated with RANKL (100 ng/mL) for 5 days. Cells on the bone slices were then removed by gentle brushing. The resorption area was evaluated by a scanning electron microscope (Hitachi S‐3700N) and quantified by ImageJ software.

### 2.6 F‐Actin Ring Immunofluorescence (IF)

To evaluate the effects of IGFBP7 on F‐actin ring formation, BMMs were cultured on bovine bone slices. Twenty four hours after the seeding, the culture medium was replaced with fresh osteoclastogenic medium with recombinant IGFBP7 (1000 ng/mL) for 5 days. After that, cells were fixed with 4% PFA, permeabilized with 0.1% Triton X‐100 and stained with rhodamine‐conjugated phalloidin (1:200, diluted in 2% BSA in PBS; Invitrogen) for 1 hour at room temperature (RT). After washing with PBS for three times, the nuclei of cells were stained with DAPI for 5 minutes at RT. Fluorescent images of F‐actin were finally captured using a fluorescence microscope (Leica, Germany). ImageJ software was used for the quantification of selected randomly images. IF analysis of p65 nuclear translocation was conducted using NIKON A1Si laser scanning confocal system.

### 2.7 Osteoblastogenesis assay

To evaluate the role of IGFBP7 on osteogenesis, MC3T3‐E1 cells from the Cell Bank of the Chinese Academy of Sciences were used. For the osteogenic differentiation, MC3T3‐E1 cells (1 × 10^4^ cells/well) were seeded in 12‐well plates and cultured in osteogenic medium (5 μmol/L L‐ascorbic acid 2‐phosphate 1 mmol/L β‐glycerophosphate), with/without different concentrations of recombinant IGFBP7, which were then maintained by replacing fresh osteogenic medium every 2‐3 days. Three days after the induction, ALP staining was performed using an ALP Activity Assay (Beyotime). To investigate the effect of IGFBP7 on mineralization, alizarin red staining (ARS) was applied at 14 days after the induction of osteogenic differentiation, using 1% solution of ARS kit (Cyagen Biosciences). The steps of ALP staining and ARS, together with the quantification, were consistent with our previous studies,^21^, ^22^ strictly following the manufacturers’ instructions.

### 2.8 Western blot analysis

To determine the protein expression of osteoclast‐related markers, BMMs (1.2 × 10^5^/well) were seeded into 6‐well plates and cultured with 0 or 1000 ng/mL IGFBP7 for 0, 1 and 3 days. To discover the underlying signalling pathways, RAW264.7 cells (5 × 10^5^ cells per well) were seeded into 6‐well plates. RAW264.7 cells were pre‐treated with vehicle or IGFBP7 (1000 ng/mL) for 6 h and thereafter exposed to RANKL (100 ng/mL) for the indicated time (0, 5, 10, 20, 30 and 60 min). Total cellular protein extracts were obtained using RIPA lysis buffer with a protease inhibitor and a phosphatase inhibitor (Beyotime). Equal amounts of protein extracts were separated by 10% SDS‐PAGE and then electroblotted to PVDF membranes (Millipore). The membranes were thereafter blocked with 5% BSA for 1 hour and then incubated with primary antibodies at 4°C overnight. After that, the membranes were probed with secondary antibodies (BOSTER) for 2 hours at 4°C. The immunoreactive bands were detected by an enhanced chemiluminescent detection reagent (Millipore) in a Bio‐Rad XRS chemiluminescence detection system (Bio‐Rad).

### 2.9 IGFBP7 overexpression and knockdown in RAW264.7 cells

Overexpression of IGFBP7 was conducted using lentivirus, and knockdown of IGFBP7 was conducted using siRNA transfection, following the methods we reported previously.^21^ In brief, lentivirus‐overexpressing IGFBP7 particles (IGFBP7‐OE group) and overexpressing control particles (Ctrl‐OE group) were obtained from GenePharma. Full‐length cDNA of IGFBP7 was prepared by PCR and then cloned into the BamHI and XhoI sites of the CMV‐MCSEGFP‐IRES retroviral vector (GenePharma). For infections, 40%‐60% confluent RAW264.7 cells were incubated with lentiviral particles and 1 mg/mL polybrene in growth medium at a multiplicity of infection of 20. After 12 hours, the infection medium was changed with fresh medium.

To knock down IGFBP7 expression in RAW264.7 cells, silencer predesigned siRNA targeting IGFBP7 (5′‐GGTATCTCCTCTAAGTAAG‐3′) (IGFBP7‐siR group) and silencer negative control siRNA (Ctrl‐siR group) were used (GenePharma). RAW264.7 cells were prepared at 40%‐60% confluence in 6‐well plates. To prepare the transfection mixture, Opti‐MEM (250 μL) containing Lipofectamine 2000 Reagent (5 μL, Thermo Fisher Scientific) was added to the Opti‐MEM (250 μL) containing siRNA (60 pmol/L). The mixture was then incubated for 20 minutes at RT, which was then added to the 6‐well plates containing 1 mL FBS‐free DMEM and incubated at 37°C. After 6 hours, the infection medium was changed with fresh medium. Knockdown of IGFBP7 expression in MC3T3‐E1 cells was conducted using the same method.

Real‐time PCR, immunofluorescence (IF) and Western blot analyses were conducted to determine the expression of IGFBP7. After that, cells were collected, and subsequent experiments were conducted.

### 2.10 NF‐κB activation luciferase reporter assay

To further study the effect of IGFBP7 on RANKL‐induced activation of NF‐κB, BMMs were transfected with NF‐κB luciferase reporter plasmid (Beyotime) following the manufacturer's protocol.^23^ After that, BMMs were pre‐treated with vehicle or the recombinant IGFBP7 (250, 500 and 1000 ng/mL) for 1 hour and then stimulated with RANKL (100 ng/mL) for 6 hours. To detect luciferase activity, BMMs were then lysed with passive lysis buffer (Beyotime). Luciferase activity was measured using the Luciferase Reporter Assay Kit (Beyotime). To explore the effect of IGFBP7 overexpression on RANKL‐induced NF‐κB activation, a luciferase reporter assay was also conducted. In brief, RAW 264.7 cells were stably transfected with control lentivirus or IGFBP7 overexpression lentivirus. After 24 hours, RAW 264.7 cells were co‐transfected with NF‐κB luciferase reporter plasmid (Beyotime) and stimulated with RANKL (100 ng/mL) for 6 hours. Luciferase Reporter Assay Kit was applied as introduced above.

### 2.11 Co‐culture of osteoblast and osteoclast precursors

To confirm the role of IGFBP7 on osteogenesis and osteoclastogenesis, a co‐culture system (24‐well Transwell chamber co‐culture system, 0.4 μm pores (Transwell) of osteoblast and osteoclast precursors was applied.^24^, ^25^, ^26^, ^27^, ^28^ 5 × 10^4^ MC3T3‐E1 cells (with/without IGFBP7 knockdown) were seeded into the upper chambers with 10% FBS/α‐MEM, and 5 × 10^4^ BMM cells were cultured in the lower chamber supplemented with osteoclastogenesis medium containing M‐CSF and RANKL, as described above. Following 5 days of co‐culture, the upper chamber was removed and osteoclastogenesis of the lower chamber was evaluated using TRAP staining.

### 2.12 In vivo experiments

All procedures of the animal experiments were approved by the Institutional Animal Care and Use Committee of the Second Affiliated Hospital, School of Medicine, Zhejiang University (Permission number: 2015‐031), strictly following the laboratory animal care and use guidelines. In total, 15 eight‐week‐old female C57BL/6 mice provided by the Academy of Medical Sciences of Zhejiang Province were used to establish an ovariectomized (OVX) murine osteoporosis model. The mice were divided evenly and randomly into three groups (n = 5 per group): Sham group, OVX group, OVX + IGFBP7 group. The details of the establishment of the OVX murine model were consistent with previous studies.^4^, ^29^ One week after the ovariectomy operation, mice in the OVX + IGFBP7 group were treated with tail vein injection of IGFBP7 (2 μg rIGFBP7 in a total volume of 100 μl PBS, once a week, based on previous studies),^17^, ^30^, ^31^, ^32^ while mice in the Sham and OVX groups were treated with PBS. After 6 weeks, all mice were sacrificed in a CO_2_ chamber. There were no deaths or side effects being observed during the 6 weeks' intervention. The distal femur specimens were collected and fixed in 4% PFA (Sigma). A random side distal femur from each mouse was scanned by micro‐CT (Scanco Medical). After 3D reconstruction, bone morphometric analysis including the bone volume/tissue volume ratio (BV/TV), trabecular thickness (Tb. Th), trabecular number (Tb. N), Conn. D and SMI was quantified and analysed. After micro‐CT analysis, all specimens were decalcified in 10% EDTA (Sigma) for 1 month and embedded in paraffin. Serial 4 µm thick sections were stained with H&E, TRAP and Masson's trichrome staining, and immunocytochemistry (IHC) staining in standard methods.^21^ Images were obtained for histomorphological analysis using a microscope (Leica).

### 2.13 Statistical analysis

Data were all presented as means ± SD. Statistical analysis was conducted using SPSS 19.0 software. All experiments were confirmed for at least three times independently, and the statistical differences were assessed using Student's *t* test and one‐way ANOVA. *P*‐value ≤.05 was considered statistically significant among groups.

## 3 RESULTS

### 3.1 IGFBP7 suppressed RANKL‐induced osteoclastogenesis in vitro

To determine the possible relationship between IGFBP7 and osteoclastogenesis, we assessed the expression of IGFBP7 during the osteoclast differentiation of BMM cells. As is shown in Figure S4A, the results of PCR analysis showed that the mRNA expression level of IGFBP7 decreased during osteoclastogenesis. Similar trend was also observed regarding the protein expression level of IGFBP7 (Figure S4B). These results indicated a possible role of IGFBP7 on osteoclastogenesis.

**Figure 1:**
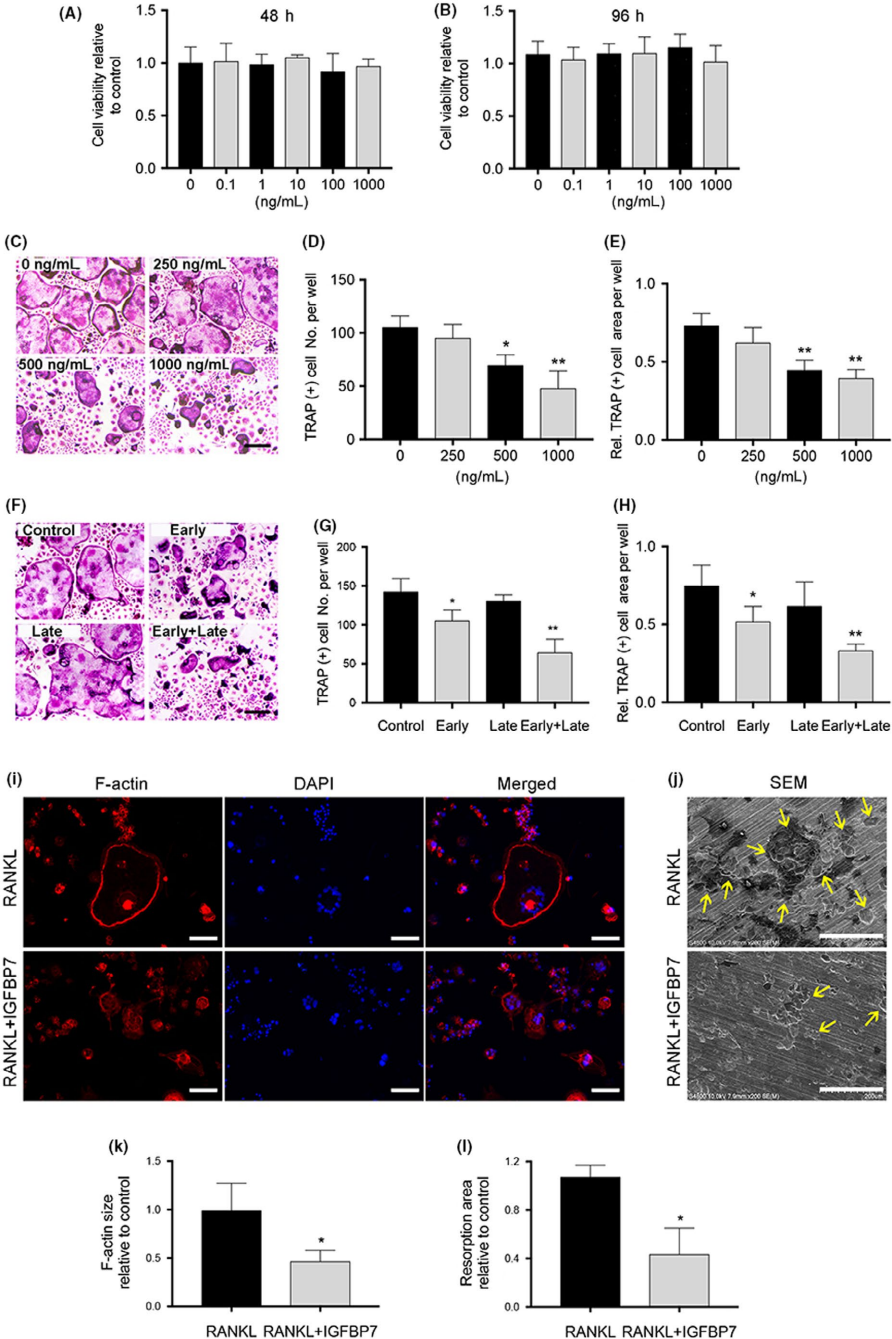
IGFBP7 suppressed RANKL‐induced osteoclastogenesis in vitro. A‐B, CCK‐8 analysis was conducted to evaluate cell viability after treated by different concentration of recombinant IGFBP7 protein for 48 and 96 h. C, BMMs were cultured with different concentration of recombinant IGFBP7 protein in osteoclastogenic medium for 5 d, and TRAP staining was performed. Scale bar = 200 µm. D‐E, The number and area of osteoclasts were calculated. F‐H, BMMs were treated with either vehicle or 1000 ng/mL recombinant IGFBP7 protein from days 1‐3 (early stage), days 3‐5 (late stage) or days 1‐5 (early + late stage) during osteoclastogenesis. The number and area of osteoclasts were calculated. Scale bar = 200 µm. I and K, Representative fluorescence images showed that recombinant IGFBP7 treatment significantly decreased the size of F‐actin ring structures. Scale bar = 100 µm. J and L, Representative SEM (scanning electron microscopy) images showed that recombinant IGFBP7 treatment decreased the area of bone resorption pits. Scale bars = 200 µm. *P < .05, **P < .01 vs the control group

We then tested the effects of recombinant IGFBP7 on the cell viability of BMMs. As is shown in Figure 1A,B, CCK‐8 analysis showed that there were no cytotoxic effects of IGFBP7 at doses 0‐1000 ng/mL on BMMs, after incubation for 48 and 96 hours below 0.8 μmol/L. To determine the effect of IGFBP7 on RANKL‐induced osteoclastogenesis, we treated BMMs with 100 ng/mL RANKL and 30 ng/mL M‐CSF in the presence of 0, 250, 500 or 1000 ng/mL of recombinant IGFBP7. As shown in Figure 1C‐E, IGFBP7 effectively suppressed the TRAP‐positive multinucleated osteoclast number and the total osteoclast area, in a dose‐dependent manner.

To further explore which particular stage of osteoclast differentiation was affected by IGFBP7, BMMs were treated with either vehicle or 1000 ng/mL recombinant IGFBP7 from days 1‐3 (early stage), days 3‐5 (late stage) or days 1‐5 (early + late stage) during osteoclastogenesis. As shown in Figure 1F‐H, IGFBP7 significantly reduced TRAP‐positive osteoclast number and size at the early stage of osteoclast differentiation, while there was little effect observed in the late‐stage group. Collectively, it can be deduced IGFBP7 could inhibit osteoclastogenesis without interfering cell viability.

### 3.2 IGFBP7 impaired F‐actin ring formation and bone resorption in vitro

To determine the effect of IGFBP7 on the osteoclasts, we first investigated its efficiency in inhibiting F‐actin ring formation, a vital prerequisite of osteoclastic bone resorption. As is shown in Figure 1I,K, typical F‐actin ring structures were observed in the RANKL‐treated control group, whereas significantly smaller F‐actin ring structures were observed in the IGFBP7 protein‐treated group. We next evaluated the effects of IGFBP7 on osteoclastic bone resorption in vitro. As is shown in Figure 1J,L, extensive and typical resorbed bone pits were observed in the RANKL‐treated control group, whereas significantly less resorbed bone pits and smaller resorption areas were observed in the IGFBP7 protein‐treated group. These data indicated that IGFBP7 inhibited F‐actin ring formation and bone resorption in vitro.

### 3.3 IGFBP7 suppressed mRNA expression of osteoclast‐specific markers

**Figure 2:**
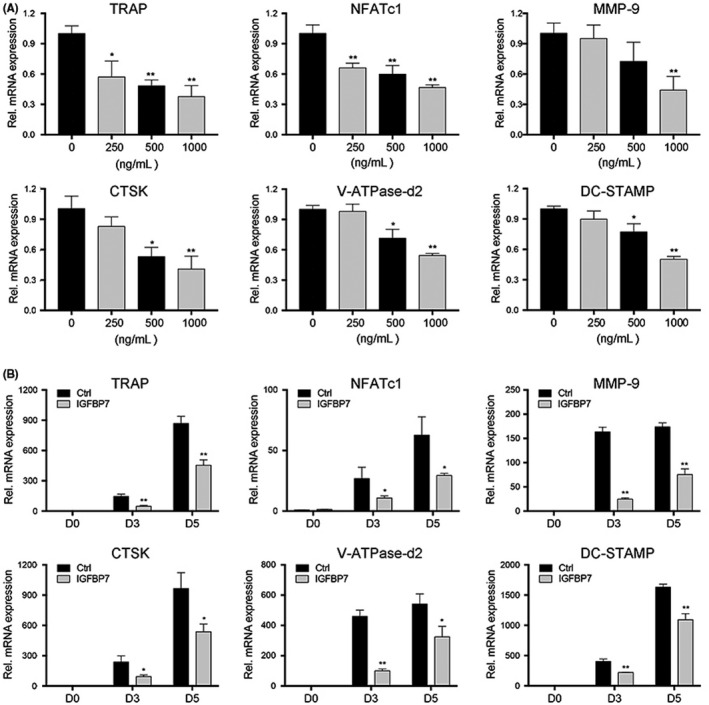
IGFBP7 suppressed mRNA expression of osteoclast‐specific markers. A, BMMs were cultured in osteoclastogenic medium for 5 d with various concentrations of recombinant IGFBP7 protein, and the mRNA expression of osteoclast‐specific markers including TRAP, NFATc1, MMP‐9, CTSK, V‐ATPase‐d2 and DC‐STAMP was determined by PCR at day 5. B, BMMs were cultured in osteoclastogenic medium with or without 1000 ng/mL recombinant IGFBP7 protein, and the mRNA expression of osteoclast‐specific markers at days 0, 3 and 5 was quantified by PCR. *P < .05, **P < .01 vs the control group

To determine the inhibitory effects of IGFBP7 on osteoclastogenesis, real‐time PCR analysis was applied to examine the mRNA expression level of osteoclast‐specific genes, including TRAP, NFATc1, MMP‐9, CTSK, V‐ATPase‐d2 and DC‐STAMP. As demonstrated in Figure 2A, recombinant IGFBP7 treatment significantly suppressed the mRNA expression levels of osteoclast‐specific genes at day 5 of osteoclastogenesis induction, in a dose‐dependent manner. Moreover, we explored the time‐dependent effect (days 0, 3 and 5 of the osteoclastogenesis induction) of IGFBP7 on osteoclastogenesis. As is shown in Figure 2B, these genes were remarkably upregulated during osteoclast differentiation for 3 or 5 days. In contrast, recombinant IGFBP7 treatment significantly suppressed their expression.

### 3.4 IGFBP7 suppressed osteoclastogenesis via inhibition of the NF‐κB signalling pathway

**Figure 3:**
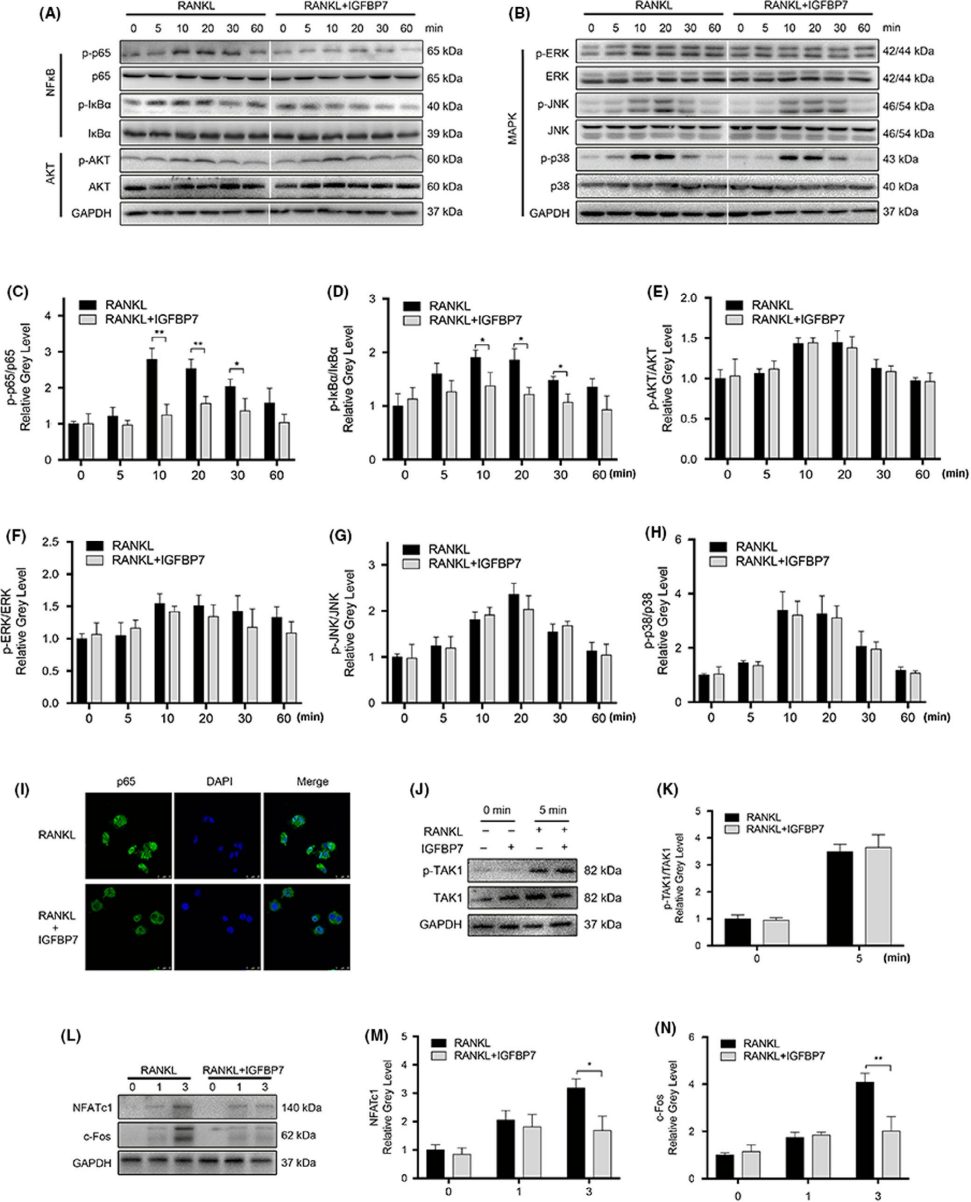
IGFBP7 suppressed osteoclastogenesis via inhibition of the NF‐κB signalling pathway. A‐H, RAW264.7 cells were pre‐treated with vehicle or IGFBP7 (1000 ng/mL) for 6 h and thereafter exposed to RANKL (100 ng/mL) for indicated time (0, 5, 10, 20, 30 and 60 min). The activation of NF‐κB, MAPKs and PI3K/AKT signalling pathways was examined by Western blot analysis and quantified accordingly. I, Representative confocal images showed that recombinant IGFBP7 treatment inhibited p65 nuclear translocation. J‐K, RAW264.7 cells were pre‐treated with vehicle or IGFBP7 (1000 ng/mL) for 6 h and thereafter exposed to RANKL (100 ng/mL) for 5 min. The activation of upstream kinase TAK1 was examined by Western blot analysis and quantified accordingly. L‐N, BMMs were cultured in osteoclastogenic medium with or without recombinant 1000 ng/mL IGFBP7 for 0, 1 and 3 d, and the expression of NFATc1 and c‐Fos was determined by Western blot analysis. *P < .05, **P < .01 vs the control group

To elucidate the underlying molecular mechanisms under the effects of IGFBP7 on osteoclastogenesis, signalling pathways involved in osteoclastogenesis were investigated. As is shown in Figure 3A‐H, recombinant IGFBP7 (1000 ng/mL) significantly attenuated p65 and IκBα phosphorylation. However, the phosphorylation level of AKT, ERK, JNK and P38 was unchanged after IGFBP7 treatment. Further IF analysis showed that IGFBP7 inhibited p65 nuclear translocation (Figure 3I).

To further explore the exact targets of IGFBP7, we assessed its effect on the phosphorylation of the upstream kinase TAK1. However, there was no significant change in the phosphorylation of TAK1 in the presence of recombinant IGFBP7 (Figure 3J,K). As NFATc1 and c‐Fos are vital transcription factors for osteoclastogenesis, we next explored the effects of IGFBP7 on their protein expression. As expected, Western blot analysis showed that RANKL‐induced expression of NFATc1 and c‐Fos was remarkably downregulated after recombinant IGFBP7 (1000 ng/mL) treatment (Figure 3L‐N).

### 3.5 IGFBP7 enhanced osteogenesis in vitro

**Figure 4:**
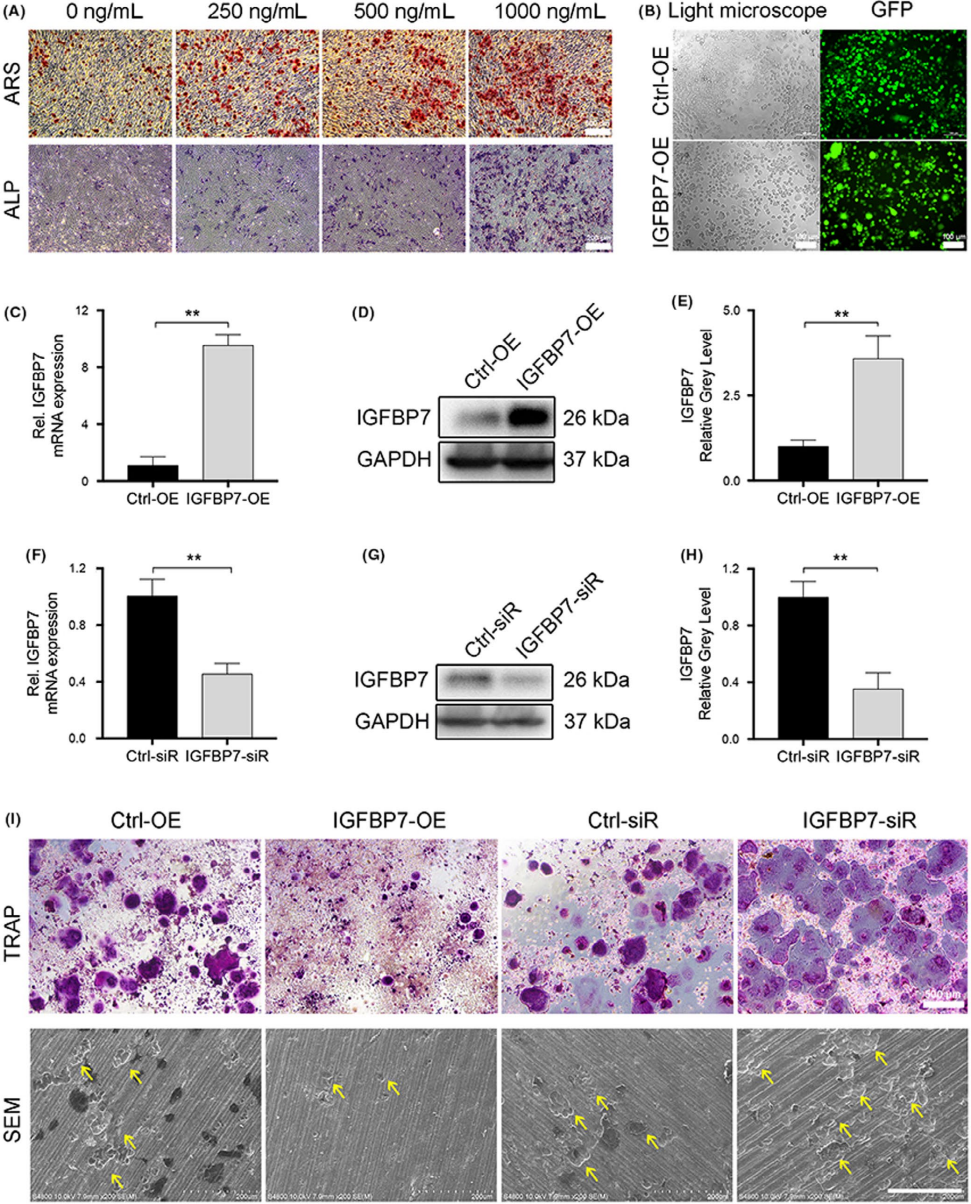
Confirmation of the effects of IGFBP7 on osteogenesis and osteoclastogenesis. A, Recombinant IGFBP7 (250, 500, and 1000 ng/mL) effectively increased the number of ALP‐positive cells and mineral depositions in a dose‐dependent manner. B, Representative light microscope and fluorescence images showed that the lentiviral vectors were efficiently applied to overexpress IGFBP7 in >80% of RAW264.7 cells. C‐H, The results of quantitative PCR and Western blotting at 3 days after the infection confirmed the successful establishment of IGFBP7 overexpression and knockdown in RAW264.7 cells. I, TRAP staining and SEM analysis showed that overexpression of IGFBP7 significantly decreased the TRAP‐positive multinucleated osteoclast number and inhibited bone resorption, vice versa. ALP, alkaline phosphatase; ARS, alizarin red staining; GFP, green fluorescent protein; OE, overexpression; SEM, scanning electron microscope; siR, siRNA. **P < .01 vs the control group

The effect of recombinant IGFBP7 on osteogenesis was explored using osteoblastic cell line MC3T3‐E1. As is shown in Figure 4A, different concentrations of recombinant IGFBP7 (250, 500, and 1000 ng/mL) effectively increased the number of ALP‐positive cells on day 3 of the induction of osteogenic differentiation. Furthermore, the results of ARS analysis showed that recombinant IGFBP7 significantly enhanced the deposition of minerals in a dose‐dependent manner. Being consistent with our previous study,^21^ these results again confirmed that IGFBP7 enhanced osteogenesis in vitro.

We also evaluated the effects of IGFBP7 on OPG and RANKL expression. In our previous study,^21^ we have shown that IGFBP7 increased the OPG expression in vivo, this time we applied additional in vitro experiments using MC3T3‐E1 cells. The results of Western blot analysis showed that different concentrations of recombinant IGFBP7 increased the protein expression of OPG, which is consistent with our previous study.^21^ Moreover, the expression of RANKL was not affected after IGFBP7 treatment (Figure S1). Taken together, these results showed that IGFBP7 increased OPG expression and enhanced osteogenesis in MC3T3‐E1 cells.

To confirm the role of IGFBP7 on osteogenesis and osteoclastogenesis, a co‐culture system of osteoblast and osteoclast precursors was applied. As is shown in Figure S5, although no statistical significance between groups was reached, there was an obvious trend that more TRAP‐positive cells were formed in the co‐culture system containing MC3T3‐E1 cells with IGFBP7 knockdown, when compared with the control group. The results of our study showed that IGFBP7 might also inhibit osteoclastogenesis through regulating the interactions between osteoblast and osteoclast precursors. Future studies are needed to confirm our results.

### 3.6 Confirmation of the effects of IGFBP7 on osteoclastogenesis using IGFBP7 overexpression and knockdown

To confirm the role of IGFBP7 during osteoclast differentiation, IGFBP7 was overexpressed in RAW264.7 cells by lentiviral particles or downregulated by siRNA. The lentiviral vectors were efficiently applied to overexpress IGFBP7 in >80% of RAW264.7 cells, which was confirmed by examining the ratio of green fluorescent protein (GFP)‐positive RAW264.7 cells using IF analysis (Figure 4B). Besides, the results of quantitative PCR and Western blotting at 3 days after the infection again confirmed the successful establishment of IGFBP7 overexpression and knockdown in RAW264.7 cells (Figure 4C‐H).

We next inducted the osteoclast differentiation of RAW264.7 cells using the same method and evaluated the role of IGFBP7 on osteoclastogenesis using TRAP staining and scanning electron microscope (SEM) analysis. As is shown in Figure 4H, the results of TRAP staining showed that overexpression of IGFBP7 significantly decreased the TRAP‐positive multinucleated osteoclast number, while IGFBP7 knockdown remarkably enhanced osteoclastogenesis. The results of SEM analysis showed that overexpression of IGFBP7 can effectively inhibit bone resorption in vitro and vice versa (Figure 4I).

**Figure 5:**
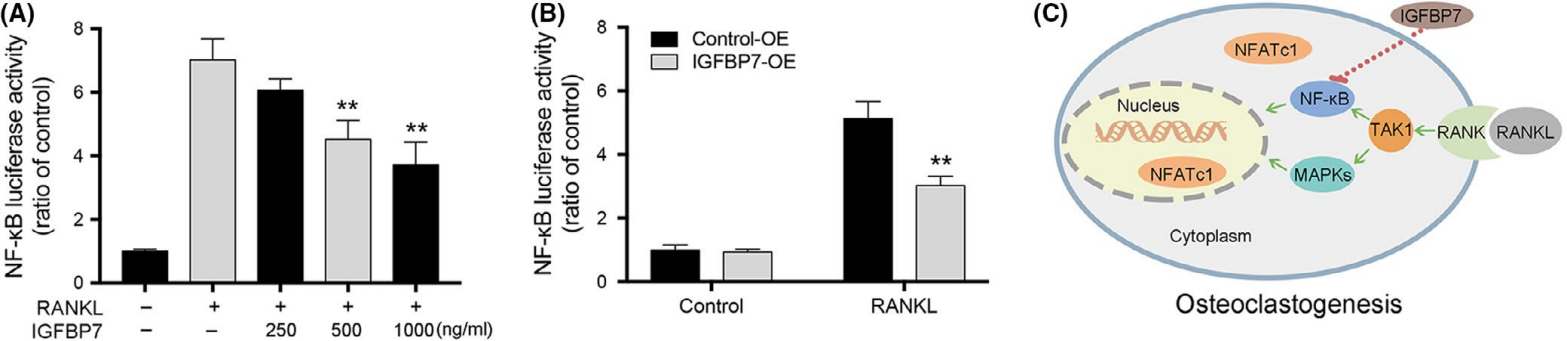
Effects of IGFBP7 on RANKL‐induced activation of NF‐κB in RAW264.7 cells. A, Transfected RAW264.7 cells were treated with various concentrations of recombinant IGFBP7. RANKL‐induced NF‐κB activation was measured with the NF‐κB luciferase reporter assay. B, NF‐κB luciferase reporter assay was also applied in RAW264.7 cells with/without IGFBP7 overexpression. Overexpression of IGFBP7 significantly inhibited RANKL‐induced NF‐κB activity. C, Schematic diagram of the possible mechanism by which IGFBP7 inhibits osteoclast differentiation and osteoclastic bone resorption. OE, overexpression. **P < .01 vs the control group

The result of NF‐κB activation luciferase reporter assay demonstrated that stimulation of BMMs with RANKL resulted in a significant increase in NF‐κB luciferase activity, which was attenuated by recombinant IGFBP7 in a dose‐dependent manner. Similar results were also confirmed by IGFBP7 overexpression in RAW264.7 cells (Figure 5A,B). In addition, we also applied IGFBP7 overexpression and knockdown in BMM cells using the same method as introduced above. The results of TRAP staining, SEM analysis and NF‐κB activation luciferase reporter assay confirmed the results we found using RAW264.7 cells (Figures S2 and S3). Taken together, these data indicated that IGFBP7 inhibited osteoclastogenesis through downregulating the NF‐κB signalling pathway (Figure 5C).

### 3.7 IGFBP7 protected against OVX‐induced bone loss in vivo

**Figure 6:**
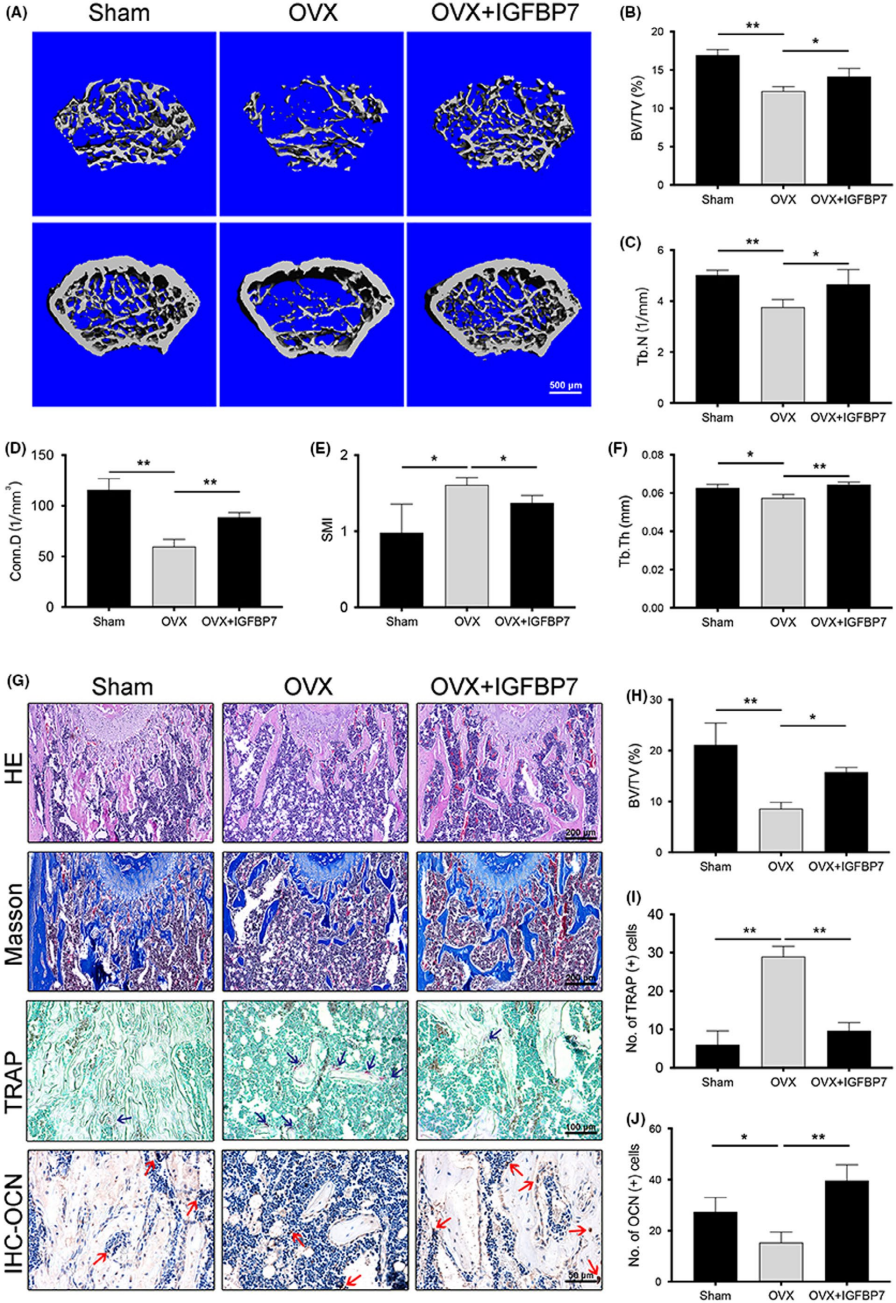
IGFBP7 protected against OVX‐induced bone loss in vivo. A, Representative 3D micro‐CT analysis images of trabecular bone of distal femur were reconstructed. Scale bar = 500 µm. B‐F, The BV/TV, Tb. N, Conn. D, SMI and Tb. Th values of micro‐CT analysis were obtained and evaluated. G, Representative images of histomorphological analysis including H&E, Masson's trichrome staining, TRAP staining and IHC staining of OCN from each group. Scale bar for H&E and Masson's trichrome staining = 200 µm. Scale bar for TRAP staining = 50 µm. H, The BV/TV (%) values of histomorphological sections were quantified using ImageJ software. I, The number of TRAP (+) cells of the TRAP staining sections was quantified. J, The number of IHC staining‐positive cells was quantified. IHC, immunocytochemistry; OCN, osteocalcin. *P < .05, **P < .01 vs the control group

To evaluate the effect of IGFBP7 on bone loss in vivo, recombinant IGFBP7 was used in a murine model of OVX‐induced osteoporosis. Radiographic and histological analyses were used to confirm the effects. The results of micro‐CT analysis showed a significant bone loss in the OVX group when compared with the Sham group, which was confirmed by BV/TV, Tb. N, Conn. D, SMI and Tb.Th (Figure 6A‐F). Together with histological analysis including H&E and Masson's trichrome staining (Figure 6G‐I), the results of in vivo analysis showed that IGFBP7 treatment (OVX + IGFBP7 group) in OVX mice remarkably reduced OVX‐induced osteoporotic bone loss.

To further determine how IGFBP7 mitigated OVX‐induced osteoporotic bone loss, TRAP staining and IHC staining of OCN were subsequently applied (Figure 6G‐J). The results of TRAP staining showed that osteoclast formation was significantly enhanced in the OVX group when compared with the Sham group. In the IGFBP7‐treated group (OVX + IGFBP7 group), the elevated osteoclast formation following OVX surgeries was significantly diminished, which was consistent with the results of micro‐CT analysis, H&E staining and Masson's trichrome staining. The results of IHC staining showed that the reduced expression of OCN in the OVX group was also diminished after recombinant IGFBP7 treatment. These results demonstrated that IGFBP7 effectively protected against OVX‐induced osteoporotic bone loss in vivo by suppressing osteoclastogenesis.

## 4 DISCUSSION

Osteoporosis is one of the most challenging ageing‐related disorders; thus, it is a major public health problem. Although current anti‐osteoporotic therapies such as oestrogen replacement therapy and bisphosphonates have been widely used, severe limitations still exist.^4^ Studies of genetic factors involved in bone development are required to identify novel therapeutic approaches, particularly genes that inhibit bone resorption and improve bone formation. The hypothesis of this study was, IGFBP7, an osteogenesis‐regulating gene that had been reported in our previous study,^21^ might play an important role in osteoclast differentiation and osteoporosis. In the present study, we showed that IGFBP7 inhibited osteoclastogenesis and bone resorption. IGFBP7 suppressed the NF‐κB signalling pathway during osteoclastogenesis. Moreover, in a mouse ovariectomy‐induced osteoporosis model, IGFBP7 treatment attenuated osteoporotic bone loss by inhibiting osteoclast activity and enhancing osteoblast activity. In conclusion, these findings indicate that IGFBP7 is a negative regulator of osteoclastogenesis via the NF‐κB signalling pathway and plays a protective role in osteoporosis. To the best of our knowledge, this is the first study to explore the effects of IGFBP7 on osteoclastogenesis and osteoporosis.

This study emphasized a novel role for IGFBP7 in regulating osteoclastogenesis and bone resorption. IGFBP7 is widely expressed in multiple human tissues and has various functions. As an important protein coding gene, studies of IGFBP7 have mainly focused on its suppressive role in various human malignancies, including thyroid carcinoma, cholangiocarcinoma, gastric cancer, hepatocellular carcinoma and breast cancer.^11^, ^13^, ^14^ However, there are reports that the effects of IGFBP7 involve insulin resistance and the risk of metabolic syndrome,^33^ which are considered closely related to the development of osteoporosis.^34^, ^35^, ^36^ More importantly, recent studies have reported that IGFBP7 may play considerable roles in bone metabolism. For example, PTH, a stimulator of osteogenesis, increases IGFBP7 expression in osteoblasts.^16^ IGFBP7 is methylated in subchondral bones and is related to its sclerosis.^37^, ^38^ IGFBP7 is also reported to be associated with the development of OA, another bone metabolism‐related orthopaedic disease.^20^ As direct evidence, our previous study showed that IGFBP7 enhanced osteogenesis in vitro and in vivo.^21^ In the present study, osteoclast formation, osteoclast function, osteoclast‐related gene expression and bone resorption together with in vivo osteoporotic bone loss were significantly inhibited by IGFBP7, which again confirmed the role of IGFBP7 in bone metabolism.

The NF‐κB signalling pathway has been shown to be required for osteoclastogenesis.^39^ RANKL‐RANK signalling is usually activated after RANKL binds to RANK and recruits TRAF6. Following the activation of RANKL‐RANK signalling, TAK1 is phosphorylated and activated, leading to the initiation of the MAPK and NF‐κB signalling pathways, followed by nuclear translocation of NF‐κB p65 to stimulate osteoclast formation. Under the stimulation of RANKL, the PI3K/AKT signalling pathway is also activated, which is partly responsible for the survival and differentiation of osteoclasts.^40^ Activation of NF‐κB signalling has also been reported to enhance osteoclastogenesis. Tzeng et al reported that Radix Paeoniae Rubra promoted osteoclastogenesis partly via activation of the NF‐κB pathway.^41^ In a mouse model, iron‐induced oxidative stress was reported to stimulate osteoclastogenesis by upregulating the NF‐κB signalling pathway.^42^ Another similar study reported that FSTL1 enhanced osteoclast formation via the activation of NF‐κB.^43^ In addition, the inhibition of the NF‐κB pathway has been widely reported as an effective approach to inhibit osteoclast formation and prevent the development of osteoporosis. Zhang et al reported that mTORC1 inhibited NF‐κB signalling and prevented osteoclastogenesis in vitro and in vivo.^44^ 4‐IPP, a macrophage migration inhibitory factor inhibitor, was also reported to suppress osteoclast formation by inhibiting the NF‐κB signalling pathway.^45^ In the present study, we found that IGFBP7 inhibited the phosphorylation of the NF‐κB signalling pathway during stimulation with RANKL. We found that IGFBP7 suppressed the phosphorylation of p65 and IκBα, and the nuclear translocation of p65, with no significant effect on the activation of TAK1. Along with the inhibition of the NF‐κB pathway, the protein expression of NFATc1 and c‐Fos was also reduced by IGFBP7. Taken together, these results indicate that IGFBP7 inhibits osteoclastogenesis through the inhibition of NF‐κB signalling.

Although there are studies indicating that the function of IGFBPs is through binding to IGFs with high affinity, thereby limiting IGF access to IGF1 receptor (IGF1R) and finally inhibiting IGF activity,^46^, ^47^ it is reported that IGFBPs also exert IGF‐independent actions.^48^ Moreover, IGFBP7 has 100 times lower affinity for IGF‐I than the other six members of this family (IGFBP1‐6).^9^, ^10^ Unlike IGFBP1‐6, so far, no definite receptor of IGFBP7 has been reported. In this study, we did not find the regulatory effect of IGFBP7 on IGF1R expression (Figure S6) and the receptors of IGFBP7 on osteoclast progenitor cells remained to be explored. Nevertheless, we do find that IGFBP7 inhibited osteoclastogenesis via inhibition of the NF‐κB signalling pathway directly or indirectly. As IGF‐I is reported to be essential for osteoclastogenesis,^49^ future studies are still needed to uncover the possible role of IGF‐I under the effects of IGFBP7 on osteoclastogenesis, and whether IGF‐I is involved during the internalization of IGFBP7 within the osteoclast progenitor cells.

Some limitations of the present study should be noted. First, although we showed that IGFBP7 effectively inhibited osteoclastogenesis in vitro and in vivo, its safety was not evaluated. As an important regulator involved in many physiological and pathological processes, an understanding of the role of IGFBP7 in humans is far from complete. Second, we reported the role of IGFBP7 on the osteogenesis of BMSCs in our previous study^21^ and the current study focused on the role of IGFBP7 on osteoclastogenesis. The effect of IGFBP7 on osteoblasts, osteocytes and BMSCs, together with their interactions during the development of OVX, was not studied. Future studies on this topic will be of interest. Similarly, future studies with additional the Sham + IGFBP7 group and positive control group using anti‐osteoclastogenesis drugs will be helpful to confirm the roles of IGFBP7 on bone metabolism. Third, we also acknowledge that we did not use transgenic or gene knockout mice, which may decrease the robustness of our conclusions. Fourth, the progress of osteoclastogenesis is very complicated, involving multiple signalling pathways. We only studied the classical pathways and not the non‐canonical pathways. The potential receptors of IGFBP7 in osteoclast progenitor cells are remained to be illuminated in future studies. In addition, in the present study, only oestrogen deficiency‐induced osteoporosis was studied; other osteoclast‐related disorders, such as inflammation‐induced osteoporosis, remain to be studied. Nevertheless, our study used both recombinant IGFBP7 protein and overexpression/knockdown to characterize the role of IGFBP7 in osteoclastogenesis. Together with our previous study, this study provides useful insight into the potential effects of IGFBP7 in regulating bone metabolism and preventing osteoporosis.

## 5 CONCLUSION

Our results show that IGFBP7 acted as a negative regulator of osteoclastogenesis by inhibiting the NF‐κB signalling pathway and played a protective role in osteoporosis. These novel insights into IGFBP7 may aid in developing potential treatment strategies for oestrogen deficiency‐induced osteoporosis and other osteoclast‐related disorders, although further studies are needed.

## CONFLICT OF INTEREST

The authors declare no conflict of interest.

## AUTHOR CONTRIBUTIONS

CY, WZ and RH: conception and design; CY, WH, MC, JL, EC, LT, KH, QD and YL: experiments and/or data analysis; CY, WH and MC: intellectual input and supervision; CY, WZ and RH: article writing with contributions from other authors.

## Data Availability

The data that support the findings of this study are available from the corresponding author upon reasonable request.
