# Supplementary material for: IGFBP7 acts as a negative regulator of RANKL‐induced osteoclastogenesis and oestrogen deficiency‐induced bone loss
Source: Cell Prolif. 2019 Dec 30;53(2):e12752. doi: 10.1111/cpr.12752 (PMC7046308; doi:10.1111/cpr.12752)
Supplement: Supplementary file 1 [file CPR-53-e12752-s001.docx]

Figure S1:


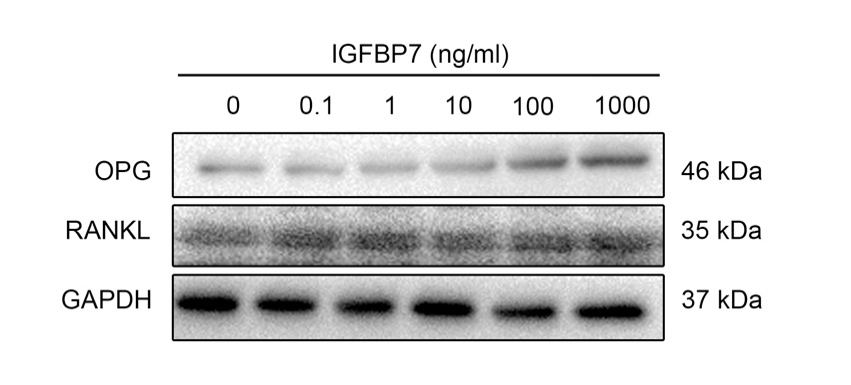


Figure S1. The results of western-blot analysis showed that different concentrations of recombinant IGFBP7 increased the protein expression of OPG in osteoblastic cell line MC3T3-E1, whereas the expression of RANKL was not affected after 3 days’ IGFBP7 treatment. OPG, osteoprotegerin; RANKL, receptor activator of nuclear factor-κB ligand.

Figure S2:


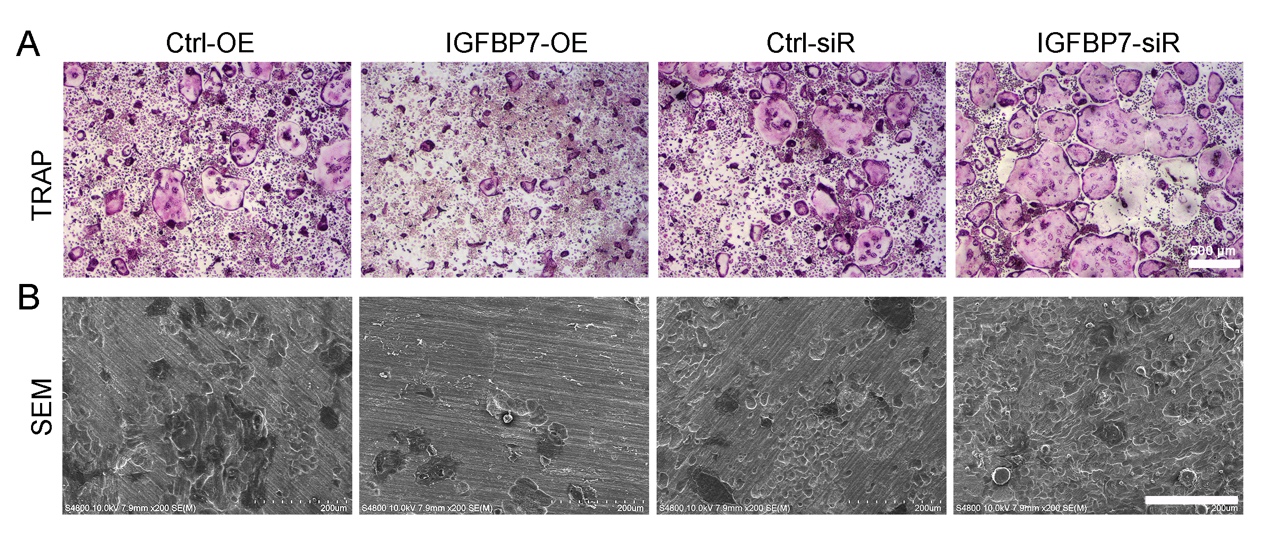


Figure S2. The results of TRAP staining and SEM analysis in BMM cells with IGFBP7 overexpression and knockdown. (A) Overexpression of IGFBP7 decreased the TRAP-positive multinucleated osteoclasts number. (B) Overexpression of IGFBP7 inhibited bone resorption, vice versa. OE: overexpression; siR: siRNA; SEM: scanning electron microscope.

Figure S3:


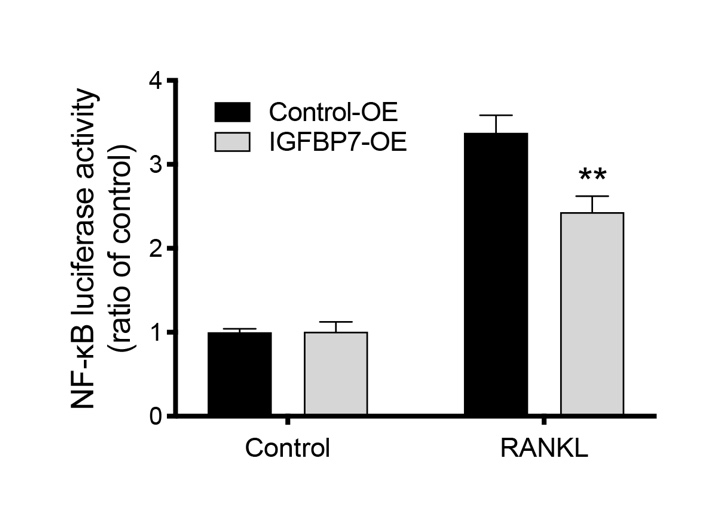


Figure S3. Results of NF-κB luciferase reporter assay in BMM cells. Overexpression of IGFBP7 inhibited RANKL-induced NF-κB activity in BMM cells. OE: overexpression. **P < 0.01 *vs.* the control group.

Figure S4:


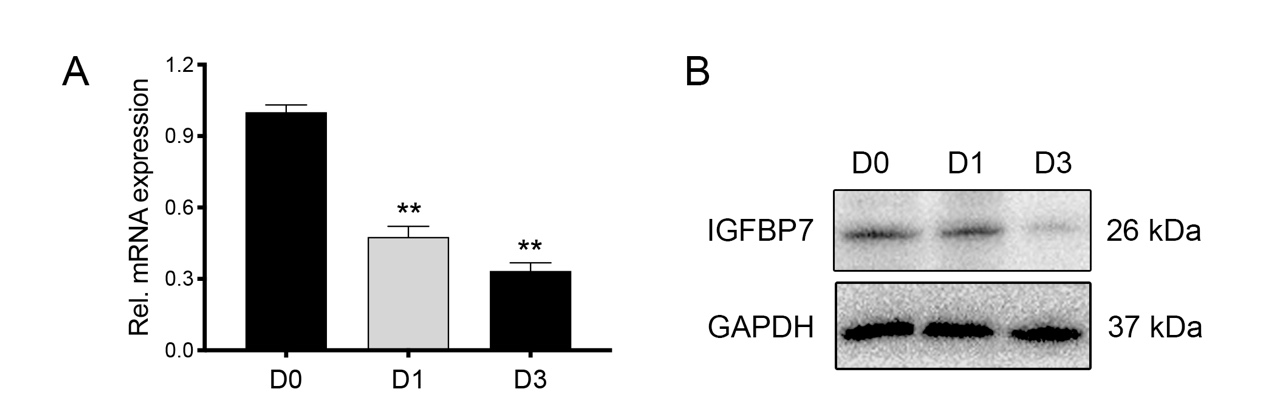


Figure S4. The mRNA and protein expression of IGFBP7 during the osteoclast differentiation of BMM cells. (A) The results of PCR analysis. (B) The results of western-blot analysis. **P < 0.01 vs. the control group.

Figure S5:


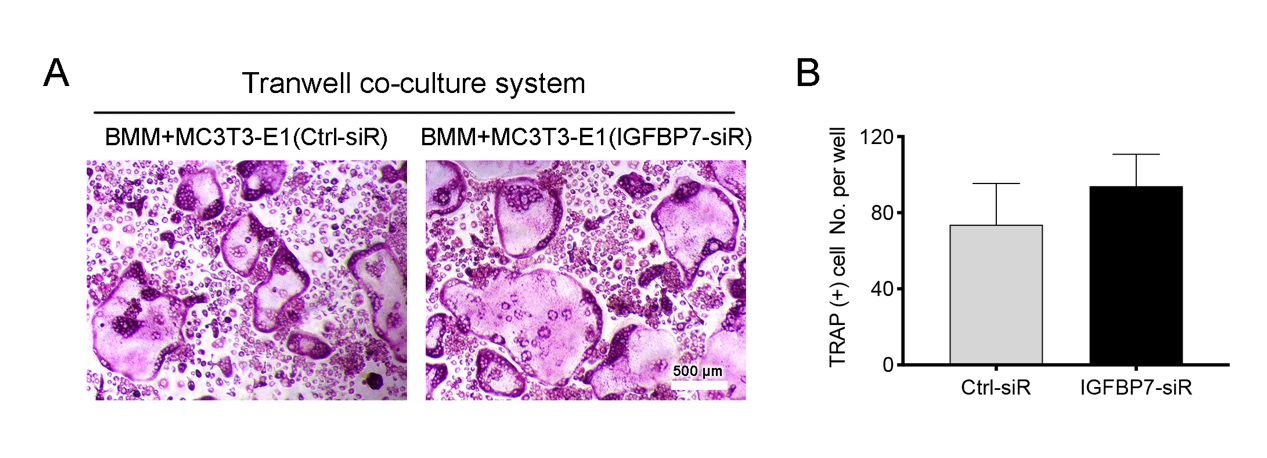


Figure S5. TRAP staining of the osteoblast and osteoclast precursors transwell co-culture system. (A) Representative TRAP staining images. (B) The number and area of osteoclasts were calculated. siR: siRNA; Ctrl: the negative control group.

Figure S6:


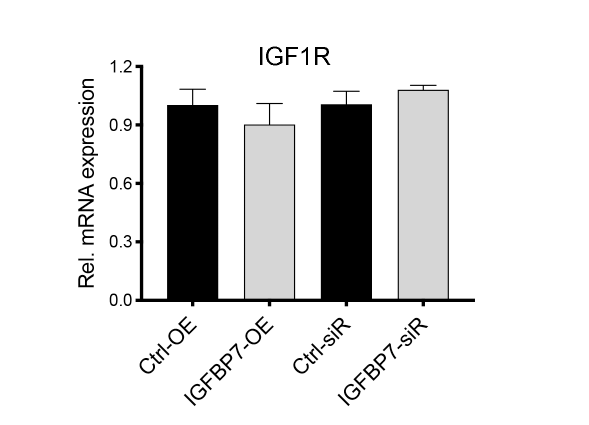


Figure S6. Results of PCR analysis in BMM cells. No significant difference regarding the IGF1R mRNA expression level was observed in BMMs when IGFBP7 was overexpressed or knocked down. OE: overexpression; siR: siRNA; Ctrl: the negative control group.
